# On the Growth of the Nails as a Prognostic Indication in Cerebral Paralysis

**Published:** 1872-06

**Authors:** S. Weir Mitchell

**Affiliations:** Transactions of the College of Physicians of Philadelphia


*On the Growth of the Nails as a Prognostic Indication in Cerebral Paralysis.* By Dr. S. Weir Mitchell. (Transactions of the College of Physicians of Philadelphia.)

During the last winter I saw Mrs. B., æt. 56, immediately after an attack of cerebral palsy. This lady had been previously in good health, and free from disease of the heart. In the evening, after dinner, while reading, she complained of headache, which increased during an hour, and was followed by sudden loss of power, complete in the left leg, and incomplete in the face and arm of the same side. There was no sensory loss, and the articulation was but slightly altered. The only remote prodrome was pain at times in the two limbs affected, but chiefly in the leg—a symptom which she ascribed to rheumatism, but which, whatever its cause, I have met with many times in persons afterwards afflicted with palsy on the same side. Without being confident of its value as a precursory symptom, I may state that in these patients there was often no rheumatic pain in the opposite side, or elsewhere.

Mrs. B. had but little intellectual disturbance, and no loss of consciousness. She slowly recovered, so as to be able to walk quite well; but the arm, after improving for two weeks, again became weaker, and finally, after some months, grew gradually well enough to be used in knitting or in holding a book. The finger-nails on the left side, which were previously healthy and rather large, became marked with deep serrations, crossing from side to side, and about one line apart. This peculiarity remained so long as she was under my care, the growth being much slower than that of the nails of the healthy arm. I regret that I did not examine the feet.

Struck by these peculiarities, I did not fail to study the nail-growth in the next case of like nature. In May, 1870, I saw a gentleman, æt. 47, the night of an attack of severe apoplexy. He was a large, ruddy person, and he also had no heart disease, but had been subject to great strain, both mental and physical. He had for a week slight defect of memory, great weariness, and occasional vertigo, with a hissing sound in the right ear, which was worse after meals. The attack came on while he was pulling off a boot at night. He fell, slightly convulsed and unconscious, and when I saw him he was able to lift the palsied hand with the other to direct my attention to it. His articulation was thick, his face strongly drawn to the left, and his tongue, when thrust out, deviated to the right side. The arm and leg were perfectly sensible, but he could stir neither of them. The leg recovered rapidly, and in a week he could walk a few steps, pushing a chair before him with the sound hand.

The morning after his attack, I stained four of the nails of the palsied right hand, down to the lower edge, with nitric acid, which tints these parts of a deep and quite lasting yellow, hoping thus to learn whether they would grow as fast as those of the other hand. To my surprise, while the left healthy nails grew as usual, the right nails did not grow at all during three weeks. Then, and while the arm was throughout still motionless, the nails began to grow, as was shown by a narrow line of white below the tinted portions. Within a week after this the fingers became controllable by the will, and gradually the whole hand and then the arm was restored, so as to perform any except the most delicate tasks required of it.

Oct. 16, I attended Mr. C., æt. 46, who was suffering from great mental excitement concerning an election in which he was interested. His previous health was perfect. While lying in bed in the evening, somewhat drowsy from a small dose of morphia, he felt his right thumb drawn into the palm; then the forearm flexed strongly on the arm, while the right face twitched slightly, and in a few moments the right arm and leg became entirely paralyzed, as to motion only. There was no deviation of tongue or face, but he could not carry either eye fully to the right side. With these symptoms there was nearly complete aphasia, his only word being "yes." On the 17th he could make negative signs with his head, although still saying "yes" to every question. On the 18th he was able to say "yes," "no," and "certainly," and could repeat after me many simple words. Oct. 20. His right hand is slightly œdematous, and he now for the first time moves his toes. His vocabulary is enlarging, but he makes the usual curious blunders, and when trying to read aloud, frequently stops at long words and miscalls them; says he understands what he hears and reads.

The temperature of the right palm was at this time $95\frac{4}{5}°$, left $97\frac{2}{5}°$ F. Ever since the attack he complained of pain in the right shoulder, and of the occasional spasmodic flexion of the thumb and fingers. At this time, the fourth day, the hand being moveless, I stained the nails, except that of the little finger, on both hands. Not the slightest growth took place on the palsied side until Nov. 2, when, seeing a line of white above the quick, I risked the prediction to his wife that within a week he would begin to move the limb. On the fourth day after, the thumb recovered some slight power, and the rest of the limb rapidly followed it, so that every muscle was under control on November 9, although for some time the extensors of the fingers moved with difficulty, because of the continued but lessening spasm of the flexors.

The whole history of these cases I have not given, thinking it enough to relate so much of them as is sufficient for my present purpose.

Perhaps, also, I ought to excuse myself for reporting but two distinct cases, in place of waiting until numerous opportunities enable me to be more sure of the constancy of the facts related. Cases of this kind, however, seen early, may not recur very often under my own eyes, and I shall probably increase the chance of studying this curious symptom by making it public.

Even now it suggests some reflections which I must be pardoned for intruding, because they will to some extent guide any future observer.

I have been unable to find that this observation has been made before. In old cerebral palsies the nails very often become deformed, and even the muscles may undergo changes which are possibly due to the neural sclerotic alterations which sometimes come on after the part has been long disused. They are then the direct result of isolation from spinal trophic influence. In recent cerebral palsies there is often œdema, but no muscular atrophy; and it is, therefore, remarkable, that the nails should ever suffer in their nutrition. It is still more curious, when we reflect that even in parts whose nerves are severed, the nails grow as usual, and that chiefly in partial nerve-wounds do we meet with clubbing or serration.

It seems as if the injury to the brain must have exerted an inhibitory influence, and the fact aids, to my mind, the view which I hold with many, that there are nutritive nerves. Theorists who follow Brown-Sequard, would probably regard the checked growth as due to a spastic contraction of the vessels feeding the nail, and as a vaso-motor nerve impression.

I cannot admit this, because no conceivable amount of such spasm could last long enough or be complete enough to cause the result without making a visible difference in the tint of the nail and the thin parts at its matrix; and these remained much as usual, perhaps even a little redder than common. To test this view, I faradized with a secondary current and dry wire brush two of the nails daily, giving great pain and intensely flushing them. They were also kept thrice a day, for a half hour, in hot water, so as to flush them as much as possible. My patient, an intelligent person, being much interested in the question, submitted readily to this treatment, but no more growth took place in these nails than in the others. I have noted the lower temperature in the last case; but in hands cut off from all nerve connection it is still slower, and yet the nails grow. It does look, therefore, in this case, as if some influence was at work here which did not act through a change in the vascular supplies. It is a point in favor of trophic nerves.

Other and most interesting questions also present themselves. The re-growth preceded the return of will power. If this should prove constant or common, it will certainly help us to answer the inevitable query as to whether the arm will recover at all, and how soon.

It is, of course, desirable to learn how often this check of nail-growth occurs—whether in all cerebral palsies, or only in certain ones—whether, in a word, it relates itself to particular brain regions, and is a direct effect, or arises from the spinal shock which these brain injuries occasion.

I trust, that I have been able to show, therefore, that this apparently trifling symptom may open the way to the solution of very important questions, and is certainly not devoid of interest for the most purely practical among us.

